# Genotoxicity of Cytolethal Distending Toxin (CDT) on Isogenic Human Colorectal Cell Lines: Potential Promoting Effects for Colorectal Carcinogenesis

**DOI:** 10.3389/fcimb.2016.00034

**Published:** 2016-03-23

**Authors:** Vanessa Graillot, Inge Dormoy, Jacques Dupuy, Jerry W. Shay, Laurence Huc, Gladys Mirey, Julien Vignard

**Affiliations:** ^1^Institut National de la Recherche Agronomique UMR 1331, TOXALIM (Research Center in Food Toxicology), Université de Toulouse, INRA, INP-ENVT, INP-EI-Purpan, Université de Toulouse III Paul SabatierToulouse, France; ^2^Department of Cell Biology, The University of Texas Southwestern Medical CenterDallas, TX, USA; ^3^Center for Excellence in Genomics Medicine Research, King Abdulaziz UniversityJeddah, Saudi Arabia

**Keywords:** cytolethal distending toxin, colorectal cancer, APC, KRAS, p53, genotoxicity, DNA double strand breaks

## Abstract

The composition of the human microbiota influences tumorigenesis, notably in colorectal cancer (CRC). Pathogenic *Escherichia coli* possesses a variety of virulent factors, among them the Cytolethal Distending Toxin (CDT). CDT displays dual DNase and phosphatase activities and induces DNA double strand breaks, cell cycle arrest and apoptosis in a broad range of mammalian cells. As CDT could promote malignant transformation, we investigated the cellular outcomes induced by acute and chronic exposures to *E. coli* CDT in normal human colon epithelial cells (HCECs). Moreover, we conducted a comparative study between isogenic derivatives cell lines of the normal HCECs in order to mimic the mutation of three major genes found in CRC genetic models: *APC, KRAS*, and *TP53.* Our results demonstrate that APC and p53 deficient cells showed impaired DNA damage response after CDT exposure, whereas HCECs expressing oncogenic KRAS^*V*12^ were more resistant to CDT. Compared to normal HCECs, the precancerous derivatives exhibit hallmarks of malignant transformation after a chronic exposure to CDT. HCECs defective in APC and p53 showed enhanced anchorage independent growth and genetic instability, assessed by the micronucleus formation assay. In contrast, the ability to grow independently of anchorage was not impacted by CDT chronic exposure in KRAS^V12^ HCECs, but micronucleus formation is dramatically increased. Thus, CDT does not initiate CRC by itself, but may have promoting effects in premalignant HCECs, involving different mechanisms in function of the genetic alterations associated to CRC.

## Introduction

The Cytolethal Distending Toxin (CDT) is a virulence factor produced by many pathogenic Gram-negative bacteria including *Aggregatibacter actinomycetemcomitans, Campylobacter* spp., *Haemophilus ducreyi, Helicobacter* species, or *Escherichia coli* (*E. coli*) (Jinadasa et al., 2011). CDT belongs to the AB_2_-type of toxin and is composed of three subunits: CdtA and CdtC represent the regulatory moiety whereas the catalytic activity relies on CdtB (Lara-Tejero and Galán, 2000). CDT exposure generally leads to cellular consequences which depend on CdtB activity, starting from cell cycle arrest to cell distention and eventually senescence or apoptosis (Johnson and Lior, 1987; Pérès et al., 1997; Gelfanova et al., 1999; Blazkova et al., 2010). CdtB shares some sequence and structural similarities with the metal-dependent phosphodiesterase family (Dlakić, 2000), and exhibits dual catalytic activities, both of which being necessary to mediate the CDT-related cellular defects. First, CdtB possesses a nuclease activity reminiscent to that of mammalian DNase I (Elwell and Dreyfus, 2000). Infection of human cells with a catalytically active CDT induces the formation of DNA strand breaks that subsequently activate the DNA damage response (Fedor et al., 2013; Fahrer et al., 2014). In contrast, CdtB displays a phosphatidylinositol-3,4,5-triphosphate (PIP3) phosphatase activity that perturbs the PI-3K/PIP3/Akt/pGSK3β signaling pathway (Shenker et al., 2007, 2016).

The first CDT-positive bacterial strains were isolated from children with gastroenteritis (Johnson and Lior, 1987). Epidemiological and *in vivo* studies support that CDT contributes to microbial pathogenicity by enhancing bacterial colonization and tissue inflammation, but the precise mechanisms still need to be elucidated (Ge et al., 2008). In mice models, infection with CDT-positive *Camplyobacter jejuni* is associated with gastritis and gastric dysplasia (Fox et al., 2004), and infection with CDT-positive *H. hepaticus* is accompanied with hepatitis and hepatic dysplastic nodules (Ge et al., 2007). These studies could be interpreted to suggest that CDT participates in the acquisition of a tumorigenic phenotype, probably through the induction of DNA damage. Actually, chronic exposure of mammalian cells with sublethal doses of CDT promotes the acquisition of cancer cells characteristics, namely genetic instability, enhanced anchorage-independent growth and defective DNA damage responses (Guidi et al., 2013). Thus, according to the growing evidence of bacterial infection associated with increased risk of cancer (Bultman, 2014), deciphering the possible role of CDT in the induction or promotion of carcinogenesis in different niches is of particular concern.

Colorectal cancer (CRC) is a leading cause of cancer-related mortality worldwide in both men and women. Sporadic cancers represent the majority of CRC cases, and only 5–10% are attributable to inherited mutations of familial cancer syndromes (Pancione et al., 2012). Genetic models of CRC identified key tumor suppressors and oncogenes whose mutations drive multiple pathways for CRC progression from healthy tissue to dysplastic adenoma and finally carcinoma (Fearon and Vogelstein, 1990). Truncating mutations in the *adenomatous polyposis coli* (*APC*) tumor suppressor is considered as one of the earliest genetic change during CRC, occurring in 70–80% cases. APC plays a role in a large spectrum of pathways such as cell adhesion and migration, cell cycle control, apoptosis, chromosome segregation, Wnt/β-catenin signaling (Fearnhead et al., 2001), and has been implicated more recently in DNA repair regulation (Narayan and Sharma, 2015). *Kirsten-ras* (*KRAS*) is a critical oncogene mutated in up to 40% of CRC (Kiyokawa and Minato, 2014). *KRAS* encodes a small GTPase and plays a key role in transduction of extracellular mitogenic signals to control cell proliferation. Finally, the tumor suppressor TP53 (p53), a multi-functional transcription factor mutated in up to 70% of CRC, regulates genes involved in cell cycle control, apoptosis, senescence and DNA repair in response to DNA damage and other cellular stresses (Toledo and Wahl, 2006).

Several parameters influence CRC, including bacterial pathogens from the gut microbiota that represent important risk factors (Allen-Vercoe and Jobin, 2014; Yu and Fang, 2015). Various bacteria have been associated with CRC including *E. coli*, the major component of the intestinal flora (Swidsinski et al., 1998; Arthur et al., 2012; Buc et al., 2013; Allen-Vercoe and Jobin, 2014; Bonnet et al., 2014). Pathogenic *E. coli* possesses numerous virulence factors important for host tissue colonization, some of which potentially may be implicated in CRC initiation or progression. Indeed, colibactin, the product of the *polyketide synthases* (*pks*) pathogenicity island, has been shown to enhance tumor multiplicity and invasion in mice models of CRC, probably through its genotoxic potential (Arthur et al., 2012). Other virulence factors from pathogenic *E. coli* have been associated with human CRC, including CDT (Buc et al., 2013; Bonnet et al., 2014). As colibactin and CDT are the two only known DNA damaging toxins produced by *E. coli*, this raises the question of the carcinogenic potential of CDT during CRC.

In this study, we analyzed the consequences of CDT-I from *E. coli* (EcolCDT) exposure on a colonic cell culture model. As we aimed to observe the possible acquisition of some hallmarks of cancer, we worked on non-transformed human colonic epithelial cells (HCEC) derived from healthy patient biopsies. These cells have been immortalized with the non-oncogenic proteins cyclin-dependent kinase 4 (Cdk4) and the catalytic component of the human ribonucleoprotein enzyme telomerase (hTERT) (Roig et al., 2010), a strategy used to immortalize various epithelial cell types without conferring tumorigenic properties. The cellular defects induced by CDT have been compared between isogenic derivative cell lines mimicking the mutation of three major genes found in CRC genetic models: loss of *APC* and *TP53*, and ectopic expression of *KRAS* (Smith et al., 2002). In the present studies these isogenically experimentally derived cells have been chronically exposed to sublethal doses of EcolCDT and analyzed for cancer hallmark acquisition. This study will allow for a better understanding of the carcinogenic potential of CDT from *E. coli* in normal or preneoplasic colonic tissues.

## Materials and methods

### Chemicals and supplements for cell-culture media

The cytolethal distending toxin from *E. coli* (CDT-I) was produced and purified in the lab at 25 mg/ml (Fedor et al., 2013) and preserved in 10% glycerol PBS (Sigma-Aldrich). Fetal Bovine Serum (FBS), puromycin, hydromycin, and zeocin were provided by Fisher Scientific. Epidermal growth factor (EGF), hydrocortisone, insulin, transferrin, sodium selenite (5 nM), and Gentamycin sulfate (50 μg/ml) were provided by Sigma-Aldrich.

### Antibodies

Anti 53BP1 (Novus Biological) from rabbit is diluted 1/3000 in PBS containing 3% bovine serum albumin (BSA), and anti γH2AX (Merck/Millipore) from mouse is diluted 1/3000. For In-Cell Western, anti γH2AX (Cell Signaling technology) from rabbit was diluted 1/200 in PBS containing 2% fetal bovine serum (FBS) and 0.2% Triton X-100 (PST buffer).

Alexa fluor 546 from rabbit and 488 (Invitrogen) from mouse were diluted 1/800 in PBS. Goat anti-rabbit antibody coupled with 770-nm fluorophore (diluted 1/1000 in PST buffer) and RedDot2 (diluted 1/1000 in PST buffer) were purchased from Biotium.

Anti β-catenin (mouse monoclonal, Santa Cruz technologies), Phalloidin-TRITC (Sigma-Aldrich) and Chicken anti-mouse Alexa 488 (Fisher scientific) were diluted 1/100, 1/10,000, and 1/2000, respectively, in PBS containing 3% BSA and 0.1% Triton X-100.

APC protein was detected by a mouse monoclonal antibody (Ab1, clone FE9) against the N-terminal end (Calbiochem) and lamins A/C with a monoclonal antibody (clone 4C11, Sigma-Aldrich). Donkey anti-mouse IgG DyLight 800 conjugate (Thermoscientific) was used as secondary antibody. Anti APC, anti lamin and secondary antibody were diluted in TBS/BSA3%/0.1% Tween (1/100; 1/10,000, and 1/20,000, respectively).

### Cell lines and maintenance

Human colonic epithelial cell (HCECs), generated and provided by Pr Jerry W Shay, were maintained on Primaria™ flask in a humidified atmosphere with 5% CO_2_ at 37°C, in 4:1 high-glucose Dulbecco modified Eagle medium/medium 199 supplemented with 2% FBS, epidermal growth factor (EGF 20 ng/ml), hydrocortisone (1 mg/ml), insulin (10 mg/ml), transferrin (2 mg/ml), sodium selenite (5 nM), and Gentamycin sulfate (50 μg/ml). In addition, CTA cells were selected by puromycin (1 μg/ml), CTR cells by hygromycin (200 μg/ml) and CTP cells by zeocin (1 mg/ml).

For HCECs chronic exposure, 25 pg/ml of CDT were added at each passage for 2–8 weeks before cells are used in additional experiments.

1CT, 1CTA, 1CTR, 1CTP were genotyped by STR profiling using the Gene Print 10 System (Promega). The gene Print 10 system is composed of nine STR loci, including *THO1, TPOX, vWA, CSF1PO, D16S539, D7S820, D13S317, D21S11, D5S818*, and the sex chromosome marker *Amelogenin (Amel)*. Amplification was done using 10 ng of template DNA applying the Gene Print 10 system following the manufacturer's recommendation. Multiplex PCR reactions were carried out by using fluorescent dye-linked primers. Labeled products were detected by electrophoretic size fractionation on an ABI 3130xl genetic analyzer (Life Technologies). The data were analyzed by using Gene Mapper ID-X software (Life technologies) to categorize peaks according to their size in relation to an internal standard run. This analysis enabled every peak to be allocated a size corresponding to the number of repeat units present. STR profiling of each cell line confirms that the cell lines are isogenic (Supplementary Table 1).

### Viability assay

Cell viability was first determined using the PrestoBlue Cell Viability Reagent (Invitrogen) according to the manufacturer's instructions. HCECs were grown on Primaria™ 96 well plates (64,000 cells/well) during 24 h. Then, cells were incubated in duplicate at three concentrations of CDT (2.5, 0.25, 0.025 μg/ml). After 72 h of treatment, cells were incubated with the PrestoBlue Cell Viability Reagent (1X) for 10 min at 37°C and fluorescence (560 nm excitation, 590 nm emission) was read using an Infinite 200 PRO reader (TECAN). The percentage of cytotoxicity was determined by comparing results with non-treated cells.

For the crystal violet proliferation assay, 6000 HCECs were grown for 24 h in 24-well plates before being treated with CDT for 5 days. After a PBS wash, cells were fixed for 10 min with 10% (vol/vol) methanol/10% (vol/vol) acetic acid at room temperature. Cells were then stained for 10 min with 1% (wt/vol) crystal violet (Sigma-Aldrich) in methanol, washed in water and the absorbed dye was released by incubation with agitation for 1 h at room temperature in methanol containing 0.1% sodium dodecyl sulfate (SDS). The dye containing solutions were then transferred to 96-well microtiter plates, and dilutions (1:2) were prepared. The optical density (OD) at 595 nm was assayed in an Infinite 200 PRO reader (TECAN).

The XCelligence system was used according to the manufacturer's instructions (Ozyme). HCECs were seeded on electronic microtiter plates (E-Plate) with 6800 cells/well. After 24 h, when Cell Index (CI) was stable, cells were treated with CDT. Cell impedance was measured in each well every 1 h for 7 days. Impedance signals were analyzed by an integrated software (RTCA Analyzer), and expressed as a CI-value that reflects cell number, cell adhesion and/or cell morphology. Acquisition and analysis was performed with the RTCA software (Version 2.0). Experiments were carried out independently in triplicate.

### Immunofluorescence

Cells were grown on glass slide in 6-well plates (100 000 cells/well) for 24 h. After 24 h of CDT treatment, cells were washed with PBS and fixed with 4% of paraformaldehyde for 20 min. Cells were washed with PBS for 5 min then permeabilized with 0.5% Triton X-100 in PBS for 15 min. Cells were blocked with 3% BSA in PBS for 1 h and then incubated with primary antibodies for 2 h. After three washes, cells were incubated with secondary antibody for 1 h and nuclei were labeled with 4,6-diamino-2-phenyl indole (DAPI).

For β-catenin and actin staining, after fixation with cold 4% paraformaldehyde, cells were permeabilized (10 min of 0.1%TritonX-100/PBS at room temperature). The saturation of non-specific fluorescence is performed in 0.1%TritonX-100/PBS/5% BSA for 15 min. Cells were incubated 2 h with mouse primary antibody at room temperature. Cells were incubated with secondary antibody (chicken anti-mouse, Alexa 488) and phalloidin-TRITC for 1 h at room temperature. Coverslips were mounted with Prolong Gold with DAPI. Slides were analyzed with a confocal laser-scanning microscope (SP8, LEICA) equipped with a 63x oil immersion objective and using 405, 488, and 565 nm lasers to reveal DAPI, Alexa 488 and TRITC dyes, respectively.

The images were analyzed using ImageJ software.

### Micronucleus assay

Cells with chronic and no chronic treatment were grown in 12-well plates (5 × 10^4^ cells/well) for 48 h. Cells were fixed with 4% of paraformaldehyde for 20 min, after a PBS wash. Then cells were permeabilized with 0.5% Triton X-100 in PBS for 15 min, and nuclei were stained with DAPI (10 nM).

### In-cell western

The In-Cell Western technique was previously described to determine genotoxicity (Graillot et al., 2012). Briefly, cells grown on Primaria™ 96-well plates (7 × 10^3^ cells/well) for 24 h. After 24 h of CDT treatment, cells were washed with PBS, and fixed with 4% paraformaldehyde for 20 min. Paraformaldehyde was then neutralized with 20 mM NH_4_Cl for 2 min and washed with PBS for 5 min. Cells were permeabilized with 0.2% Triton X-100 in PBS for 5 min and washed with PST buffer. Cells were blocked with MAXblock Blocking Medium (Active Motif, Belgium) with PHOSSTOP (Roche) for 60 min at room temperature followed by incubation with the primary antibody in PST buffer for 2 h. After three washes in PST, secondary detection was carried out using an infrared fluorescent dye conjugated antibody absorbing at 770 nm. For DNA labeling, RedDot in PST was used in combination with the secondary antibody. After 1 h incubation and three washes in PST, the DNA and the γH2AX were simultaneously visualized using an Odyssey Infrared Imaging Scanner (Li-Cor ScienceTec) with the 680 nm and the 770 nm fluorophores. Relative fluorescent units for γH2AX per cell were divided by the fluorescence per cell of the control vehicles to determine the modification in H2AX phosphorylation compared with the control. All experiments were carried out independently in triplicate. The positive control used was etoposide at 10 μM.

### Western blot analysis

HCEC 1CT and CTA (shRNA-mediated APC knockdown) cells were washed in ice-cold PBS, scrapped and pelleted by centrifugation. The whole lysates were collected in electrophoresis sample buffer containing 50 mM Tris–base/150 mM Nacl (pH 7.5), 1% Triton X100, 2% sodium deoxycholate, and 2% sodium dodecyl sulfate. Phenylmethylsulfonyl fluoride (10 mM), dithiothreitol (10 mM), and protease cocktail inhibitors (1:100) were added extemporary. Lysates were further homogenized by sonication on ice, and heated at 70°C for 10 min. Protein concentrations were measured by the Lowry Assay (Bio-Rad Lab). Protein (50 μg/well) was loaded on Nupage 3–8% Tris-acetate precast gels (Life Technologies, St Aubin, France). The proteins were separated after 90 min (130 V continuous) by using 1X Tris acetate SDS running buffer (Life Technologies). The proteins were transferred and blotted onto polyvinylidene fluoride membranes (Fisher Scientific) with Nupage transfer buffer (1X) supplemented with 10% methanol + 0.1% SDS (overnight at 4°C, 15V constant), and blocked in Tris-Buffered Saline (TBS)/BSA 3%/Tween 0.1% for 1 h. The membrane was first probed with primary antibodies overnight at 4°C, washed with TBS 1X 0.1% Tween and treated with the infrared dye-conjugated secondary antibody for 1 h. The fluorescent proteins were visualized using an Odyssey infrared Imaging Scanner (Li-Cor ScienceTec, les Ullis, France) with 680 and 770 nm fluorophores.

### Soft agar clonogenicity assay

In a 6-wells plate coated with a lower layer media containing Noble agar 0.5%, cells were seeded in medium 0.375% Noble agar at 5000 cells per well, in triplicate. After 20 days, colonies larger than 100 μm in size were counted in the whole well. Experiments were performed in three independent experiments.

### Statistical analysis

The results (*n* ≥ 3) were analyzed using software GraphPad Prism 4 for Windows. Different responses of treatments and genotype effect were analyzed by one-way ANOVA (statistical differences indicated by dollars “$” in the figures) and Student's *t*-test (statistical differences indicated by asterisks “*” in the figures). When ANOVA showed a statistically significant effect (*p* < 0.05), comparison among data was done using Tukey's HSD *Post-hoc* test.

## Results

**Figure 1:**
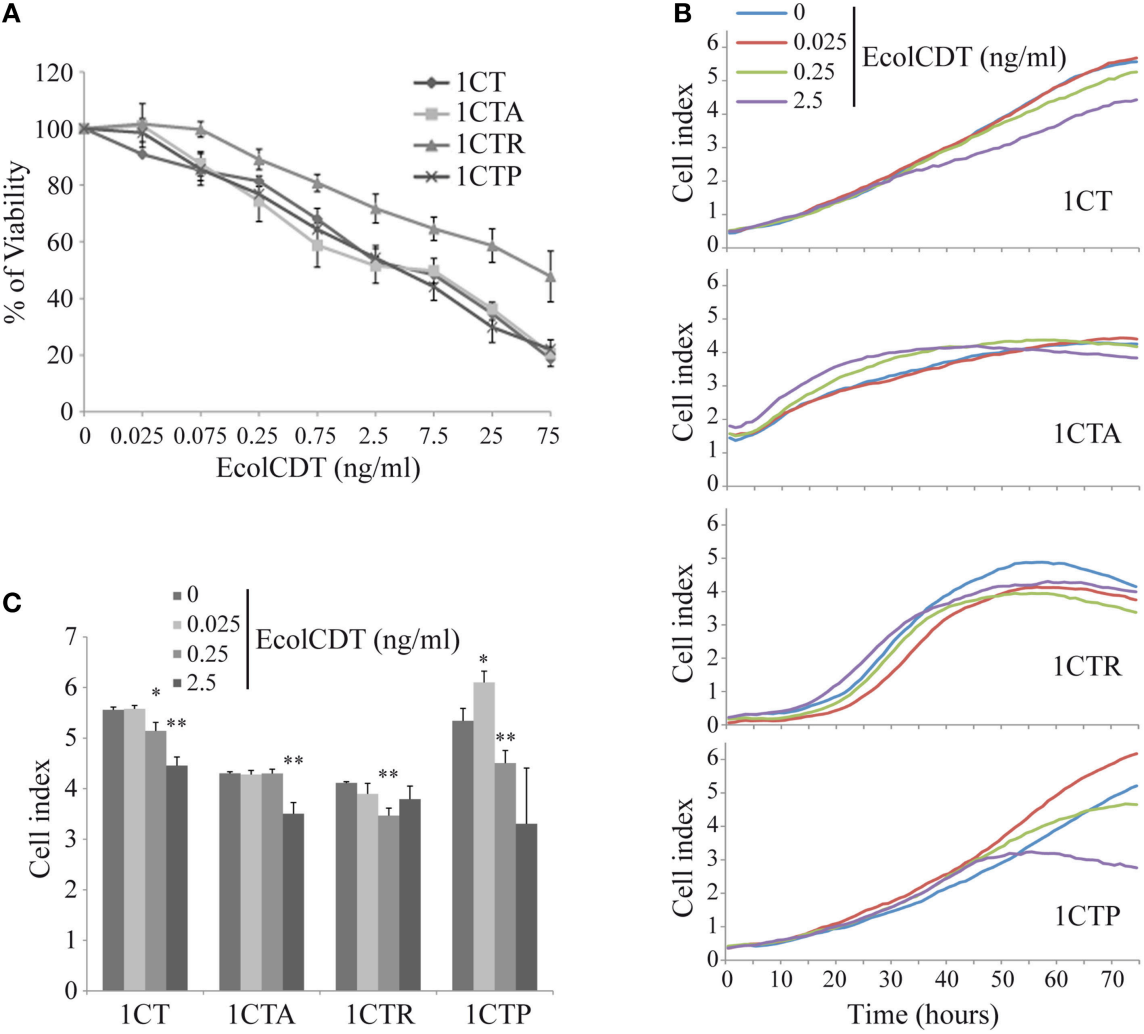
**EcolCDT exposure affects cell viability of human colonic epithelial cells. (A)** 1CT, 1CTA, 1CTR, and 1CTP human colonic epithelial cells (HCECs) were cultured for 3 days in the presence of different doses of EcolCDT (indicated above the graph). Cell viability was assessed using the PrestoBlue Cell Viability Reagent. Results present the mean ± SEM of at least three independent experiments. **(B)** Cell Index was measured in real-time and representative cell growth curves of the first 76 h of CDT treatment are shown. **(C)** The Cell Index-values at 76 h post-treatment are represented. Results represent the mean ± *SD* of three independent experiments; statistical differences were analyzed by a Student's *t*-test between the indicated conditions (*P < 0.05; **P < 0.01).

To study the possible cellular impact of EcolCDT intoxication in the colon, immortalized non-transformed adult HCECs termed 1CT (“C” for CDK4 and “T” for Telomerase) have been employed (Roig et al., 2010). Moreover, three isogenic cell lines obtained from 1CT cells have been used to model different genetic pathways to CRC (Smith et al., 2002). Two of these cell lines, either expressing the KRAS^V12^ oncogene or an shRNA directed against p53 (termed 1CTR and 1CTP, respectively), have already been characterized (Eskiocak et al., 2011). The third one, termed 1CTA, has been obtained by shRNA-mediated downregulation of APC (Supplementary Figure 1). The down-expression of APC (Supplementary Figure 1A) leads to the constitutive activation of β-catenin (nuclear translocation, Supplementary Figure 1B) and the actin network disruption (phalloidin staining, Supplementary Figure 1C). Firstly, the EcolCDT cellular sensitivity was analyzed by the PrestoBlue viability assay. The 1CT cell line and its three mutated derivatives show a dose-dependent decrease of cellular viability with toxin doses from 25 to 75 ng/ml (Figure 1). The 1CT, 1CTA, and 1CTP cell lines exhibit similar CDT sensitivities with approximately the same LD50 (2.5 ng/ml; Lethal Dose 50, killing 50% of cells in after 72 h of EcolCDT treatment). However, 1CTR cells were more resistant and show a higher LD50 (75 ng/ml). These results have been further confirmed by the crystal violet cell proliferation assay (**Figure 4A**) after 5 days of CDT exposure. Finally, we followed in real time the cell index (CI) by XCelligence technology, measuring the impedance. CI is an arbitrary unit that reflects the number of cells, cellular adhesion and shape. After seeding, cells were treated with EcolCDT for 7 days. In Figure 1B are shown representative curves of CI evolution after treatment of each cell line. The Figure 1C presents the stabilized cell index after 76 h of treatment of three independent experiments. The 1CT and 1CTP exhibit a slower rate of CI increase after CDT exposure, according to a dose-dependent manner (Figure 1B). Then, the stabilized CI at 76 h reflects this tendency (Figure 1C). Of note, at the lowest dose, 1CTP has a higher CI than untreated cells, which could be explained by an increase of cell number, cell adhesion or distension. 1CTA cells are sensitive at the highest dose, with a faster rate of CI increase to reach a lower CI when stabilized. In contrast, 1CTR cells appear more resistant, with a decrease in CI after 76 h of CDT at 0.25 ng/ml but not at 2.5 ng/ml (Figure 1C). According to these data, each cell line exhibits singular growth rates that are differentially impacted by CDT treatments, pointing out specific responses to CDT for the four HCECs that cannot be evaluated by a basic viability assay. To conclude, normal HCECs are sensitive to CDT intoxication, and KRAS^V12^ introduction and expression confers partial resistance to EcolCDT cytotoxicity.

**Figure 2:**
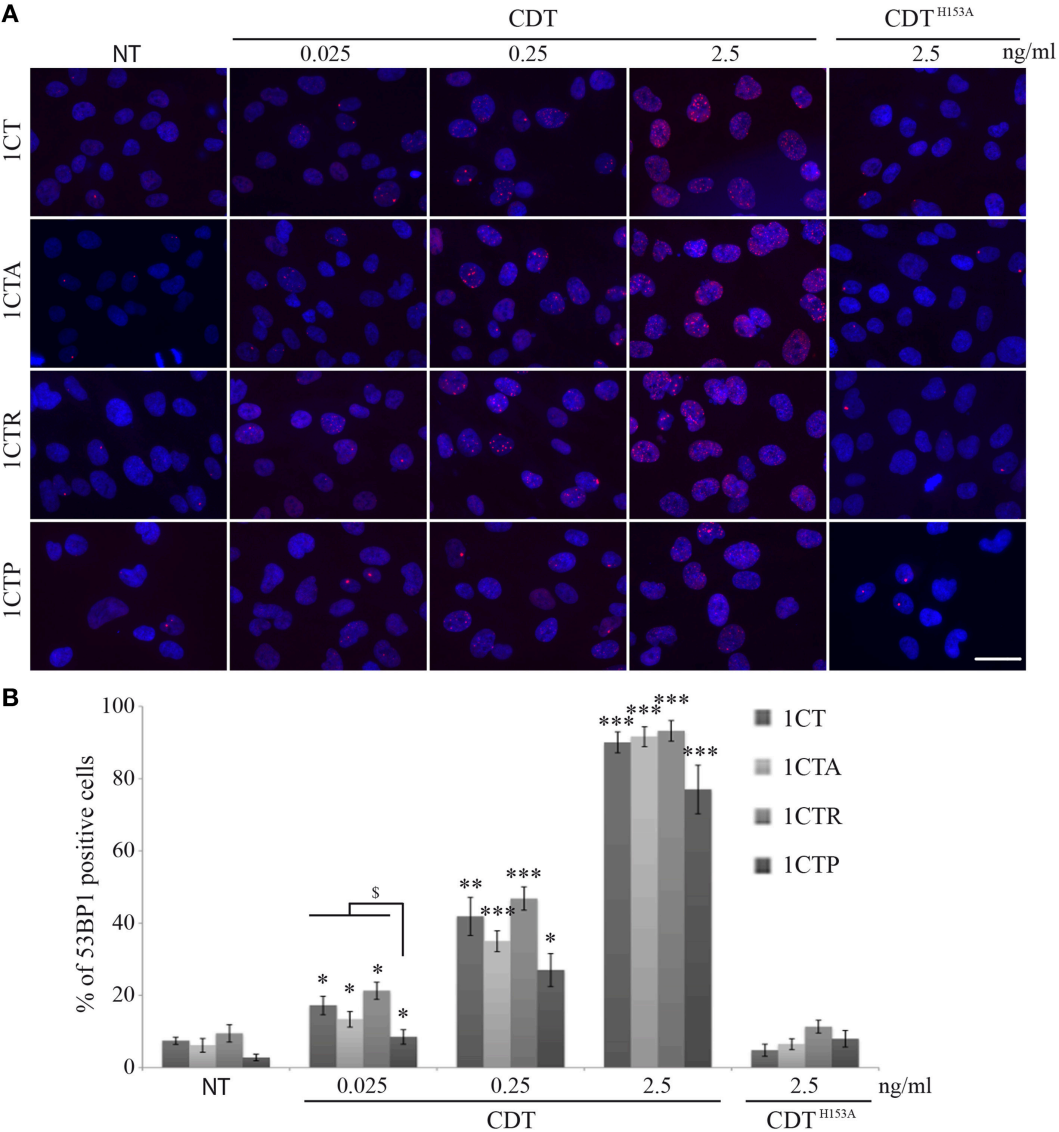
**EcolCDT exposure induces the 53BP1 recruitment to sites of DNA damage in HCECs. (A)** Representative images of 53BP1 immunostaining (red) in HCECs cells treated with the indicated doses of wild-type CDT or a catalytically dead mutant (CDT^*H*153*A*^) for 24 h. DNA was stained with DAPI (blue). NT represents non-treated cells. Scale bar = 50 μm. **(B)** Quantification of HCECs positive for 53BP1 presented in **(A)**. Cells were scored positive when containing more than five 53BP1 foci. Results represent the mean ± SEM of at least four independent experiments; statistical differences were analyzed by a Student's *t*-test (indicated by asterisks) between the indicated conditions, or by one-way ANOVA (indicated by dollars) for multiple comparisons, followed by Tukey's HSD *post-hoc* test (* or $ *P* < 0.05; **P < 0.01; ***P < 0.001).

Many studies have pointed out the ability of CDT to induce genomic DNA double strand breaks (DSBs) in infected cells (Jinadasa et al., 2011). Therefore, we investigated DSB formation induced by EcolCDT in the 1CT cell line and its isogenic derivatives. Firstly, the proportion of DSB-accumulating cells after CDT exposure has been quantified by immunofluorescence studies using an antibody directed against 53BP1, a well-defined DSB biomarker that form nuclear foci at DSB sites (Vignard et al., 2013). A 24 h CDT treatment leads to a dose-dependent increase of damaged cells in the four cell lines (Figures 2A,B). Indeed, even an exposure to a very low dose of EcolCDT, considered as subtoxic (25 pg/ml, see Figure 1), induces a slight increase of DSB-accumulating cells, which is statistically higher compared to untreated cells in each cell line (Figure 2B). At 2.5 ng/ml of CDT, almost all cells exhibit 53BP1 foci (Figures 2A,B). In contrast, cells exposed to 2.5 ng/ml of an inactive form of CDT (CDT^H153A^), composed by a CdtB catalytic mutant (Fedor et al., 2013), do not accumulate DSB (Figures 2A,B). When comparing the different cell lines, only 1CTP displays statistical differences, accumulating less damaged cells at 25 pg/ml of CDT, compared to 1CT (Figure 2B). Based on these data, 1CT and its derivatives show a dose-dependent augmentation of cells accumulating DSB in response to EcolCDT, with 1CTP cells being less impacted.

**Figure 3:**
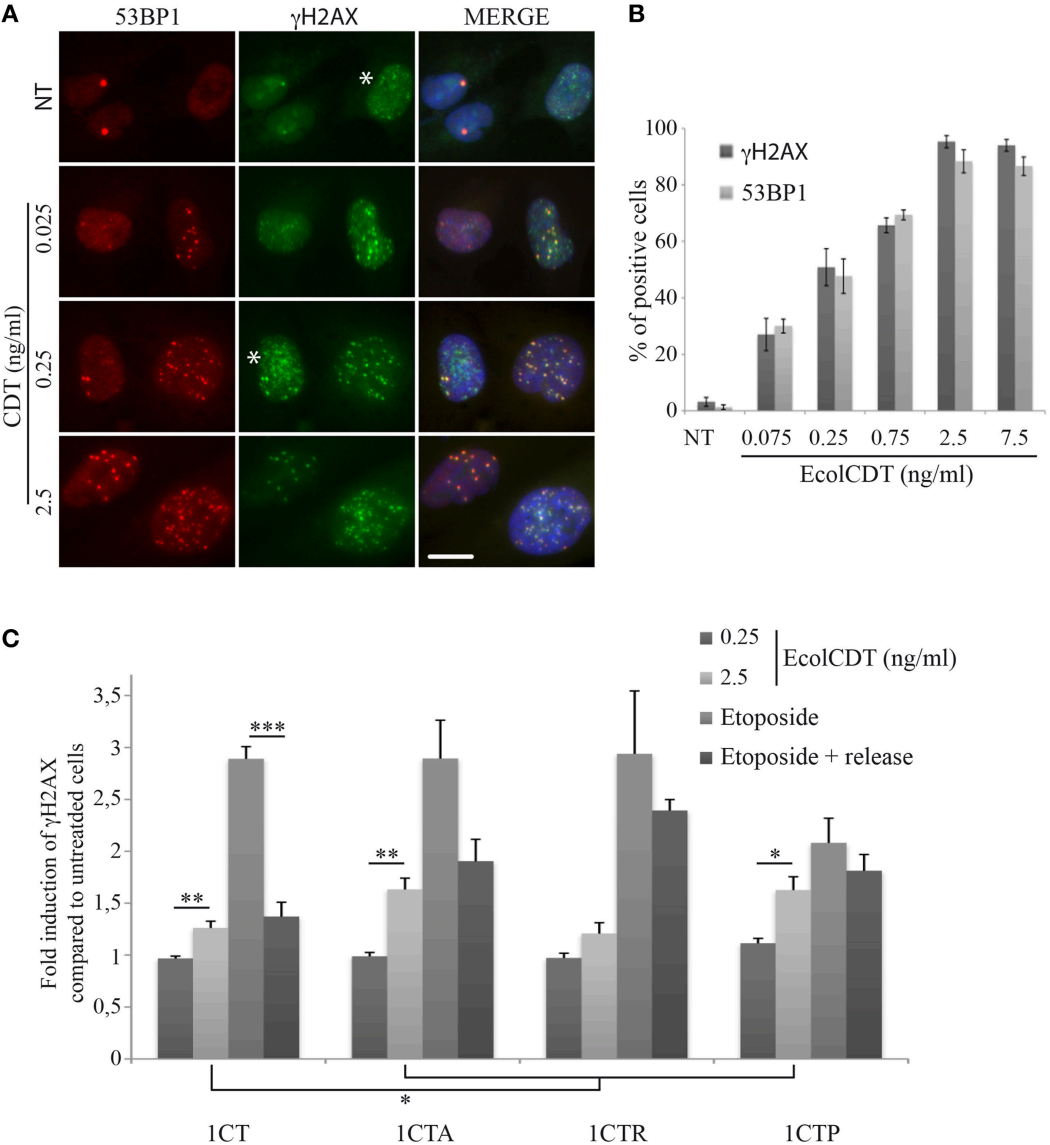
**EcolCDT exposure induces γH2AX increase in HCECs. (A)** Representative images of 53BP1 immunostaining (red) and γH2AX (green) in HCECs cells treated with the indicated doses of wild-type CDT for 24 h, showing the colocalization of 53BP1 and γH2AX foci (MERGE). DNA was stained with DAPI (blue). NT represents non-treated cells. White asterisks marks cells with γH2AX staining unrelated to DSB. Scale bar = 20 μm. **(B)** Quantification of HCECs positive for γH2AX or 53BP1 presented in **(A)**. Cells were scored positive when containing more than five foci. Results represent the mean ± SEM of at least four independent experiments. **(C)** In-Cell Western of γH2AX on HCECs. HCECs were treated with the indicated doses of EcolCDT for 24 h, or treated with 10 μM of etoposide and released or not in fresh media. Results represent the mean ± SEM of at least four independent experiments; statistical differences were analyzed by a Student's *t*-test between a condition and the non-treated cells or between the indicated conditions (**P < 0.01; ***P < 0.001).

Next, to obtain more insight on the response to DNA damage in the four isogenic cell lines, the phosphorylation of H2AX on Ser139 (γH2AX), the most common DSB biomarker (Vignard et al., 2013), was evaluated by In-Cell Western strategy (Graillot et al., 2012). The use of two different DSB biomarkers (i.e., 53BP1 and γH2AX) has been validated in 1CT cells by co-immunofluorescence analyses, showing that both signals display same patterns of positive cells after treatments with increasing doses of EcolCDT (Figures 3A,B). However, the γH2AX background signal is slightly higher compared to 53BP1, probably corresponding to replicative stress signaling (Figure 3A, see asterisks). Compared to immunofluorescence observations, no γH2AX signal increase can be detected by In-Cell Western when cells are exposed to 250 pg/ml of EcolCDT during 24 h (Figure 3C). This result suggests that the basal γH2AX level of the HCEC 1CT lineage does not permit to detect the CDT-induced γH2AX enhancement by In-Cell Western, this technique allowing global γH2AX signal detection. However, at 2.5 ng/ml of CDT, the γH2AX signal shows a 1.26-fold increase in 1CT, 1.63-fold in 1CTA, 1.21-fold in 1CTR, and 1.63-fold in 1CTP compared to the respective control cells, that is statistically different from the untreated condition, except for 1CTR. Moreover, the γH2AX signal increase seems higher in 1CTA and 1CTP cells compared to 1CT, even if no statistical difference can be observed (*P* = 0.104 for 1CTA and *P* = 0.0746 for 1CTP). This suggests that the DSB-repair machinery may be more efficient in 1CT cells compared to 1CTA and 1CTP cells after induction of DNA damage.

To test this hypothesis, cells were exposed to the DSB-inducing agent etoposide for 2 h or released in fresh media for a 16 h recovery time after 1 h of etoposide. The DSB-repair capacity has been evaluated by the reduction of the γH2AX signal between these two conditions. After 2 h of etoposide, the γH2AX signal is enhanced almost three times compared to the basal level for 1CT, 1CTA, and 1CTR, while it is only enhanced two times for 1CTP, supporting that the detection and signaling of DSBs are similar between 1CT, 1CTA, and 1CTR. When 1CT cells are released for 16 h in fresh media after etoposide, the γH2AX signal drops to only 1.37-fold increase compared to control cells, which is statistically different from the etoposide treatment without recovery time (*P* < 0.0001) but not from untreated cells (*P* = 0.054). Thus, 1CT cells seem to efficiently repair DSBs. After etoposide release, 1CTA cells also displays some DSB repair capacity but less compared to 1CT: γH2AX signal represent 1.90-fold increase compared to the basal level (statistically different; *P* < 0.05) and is not statistically different from etoposide for 2 h (*P* = 0.057). The 1CTR situation is comparable to that of 1CTA, with even less efficient DSB repair: 1CTR cells released from etoposide still exhibit a 2.39-fold increase in γH2AX signal that is close to the 2.94-fold increase for etoposide without release (*P* = 0.44). Finally, 1CTP cells are defective in DSB signaling because the γH2AX level after 2 h of etoposide is statistically lower than for 1CT cells (*P* < 0.05). Furthermore, 1CTP are also defective in DSB repair because the γH2AX signal increase is almost the same between etoposide 2 h and etoposide with release (2.08- and 1.81-fold, respectively; *P* = 0.36).

Taken together, these data reveal that the higher γH2AX induction in 1CTA and 1CTP compared to 1CT after EcolCDT is probably due to impaired DNA damage response, as revealed by etoposide treatments. 1CTR cells, however, seem defective in DSB repair but do not appear more damaged than 1CT after CDT.

**Figure 4:**
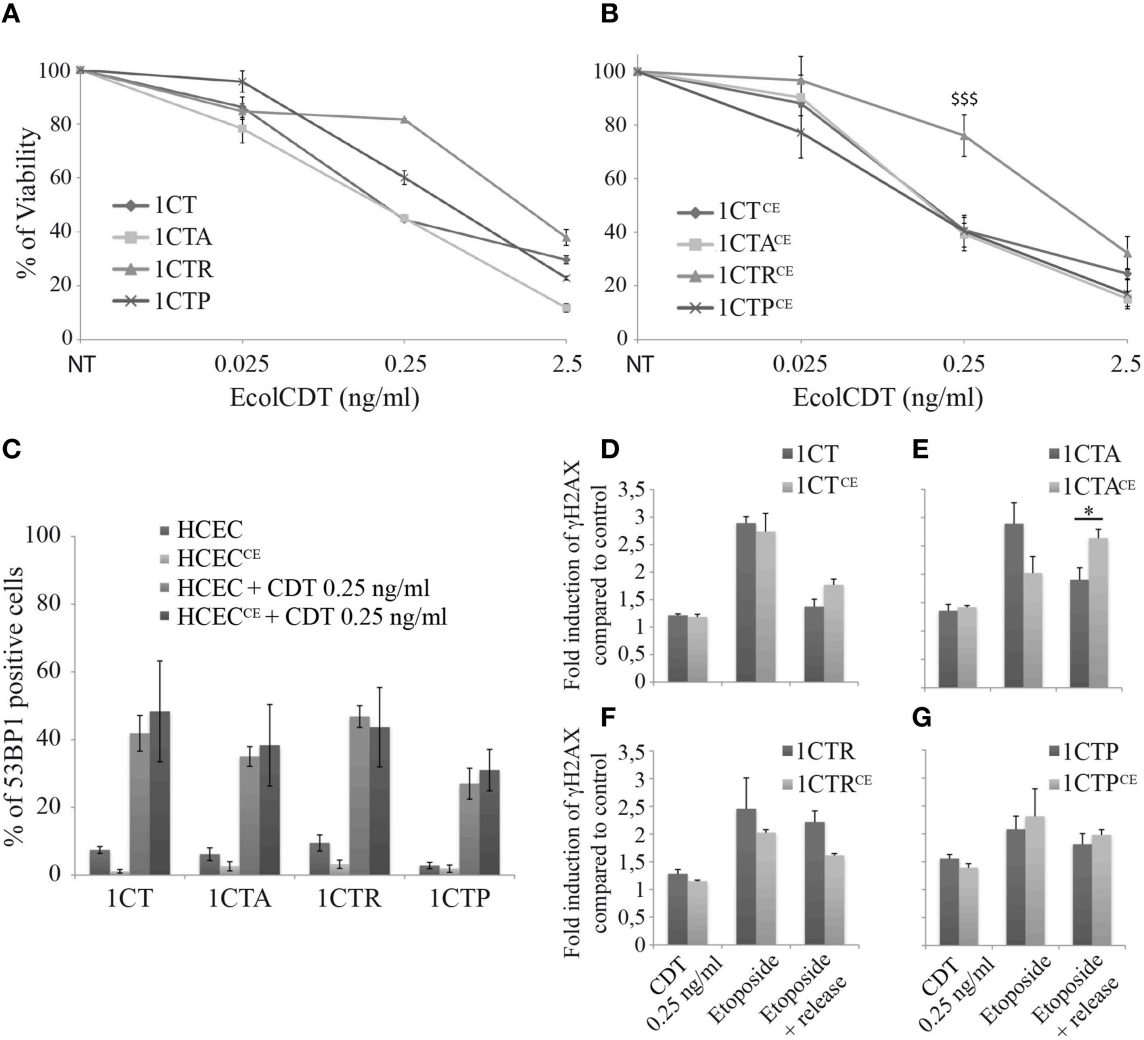
**Comparative analyses of HCECs chronically exposed or not to EcolCDT. (A,B)** HCECs **(A)** and HCECs^CE^ **(B)** were exposed for 5 days to EcolCDT and cell viability was determined by crystal violet staining. NT represents non-treated cells. Results present the mean ± SEM of at least three independent experiments; statistical differences were analyzed by one-way ANOVA followed by Tukey's HSD *post-hoc* test ($$$*P* < 0.001) **(C)** Quantification of HCECs and HCECs^CE^ positive for 53BP1 after a 24 h treatment with EcolCDT. Cells were scored positive when containing more than five 53BP1 foci. Results represent the mean ± SEM of at least three independent experiments. **(D–G)** In-Cell Western of γH2AX on chronically exposed (HCECs^CE^) or not (HCECs) 1CT **(D)**, 1CTA **(E)**, 1CTR **(F)**, or 1CTP **(G)**. HCECs were treated with 0.25 ng/ml of EcolCDT for 24 h, or treated with 10 μM of etoposide and released or not in fresh media. Results represent the mean ± SEM of at least three independent experiments; statistical differences were analyzed by a Student's *t*-test between the indicated conditions (*P < 0.05).

In mouse, chronic infection with CDT-producing bacteria has been associated with dysplasia, highlighting a possible role for CDT during carcinogenesis (Fox et al., 2004; Ge et al., 2007). To address the question of EcolCDT contribution in acquisition of tumorigenic properties in normal or premalignant HCECs, we chronically exposed 1CT, 1CTA, 1CTR, and 1CTP cells to a sublethal dose of CDT (25 pg/ml) for up to 8 weeks. These cells (termed 1CT^CE^, 1CTA^CE^, 1CTR^CE^, and 1CTP^CE^) display similar dose-responses compared to non-chronically exposed cells in crystal violet proliferation assay (Figures 4A,B), with 1CTR^CE^ cells being more resistant (*P* < 0.0001). In the same way, the proportion of cells suffering DSBs after a 250 pg/ml CDT treatment for 24 h, assessed by the counting of 53BP1 positive cells during immunofluorescence observations, does not vary between 1CTs and 1CTs^CE^ cells (Figure 4C).

Then, In-Cell Western studies were conducted to analyze the DSB repair capacities of the chronically exposed cells (Figures 4D–G). Similar to 53BP1 immunofluorescence observations, the γH2AX induction after 2.5 ng/ml of EcolCDT for 24 h does not differ with or without chronic exposure for the four cell lines. Moreover, similar responses to etoposide were obtained when comparing 1CT and 1CT^CE^ (Figure 4D) or when comparing 1CTP and 1CTP^CE^ (Figure 4G). However, 1CTA^CE^ cells show drastic changes in response to etoposide compared to 1CTA (Figure 4E), especially after the recovery time. Indeed, albeit the γH2AX induction decreased to 1.90-fold the basal level in 1CTA, it remained to 2.64-fold the basal level in CTA^CE^, which is statistically different (*P* < 0.05). Thus, chronic exposure to CDT seems to perturb the DSB repair capacity of 1CTA cells. The results for 1CTR are misleading, because the γH2AX induction appears diminished after release from etoposide when comparing 1CTR to 1CTR^CE^ (Figure 4F), but no statistical difference were observed (*P* = 0.055). To conclude, chronic exposure to CDT does not appear to specifically modify the cellular response to CDT but may rather alter the global response to DNA damage, especially in 1CTA cells.

**Figure 5:**
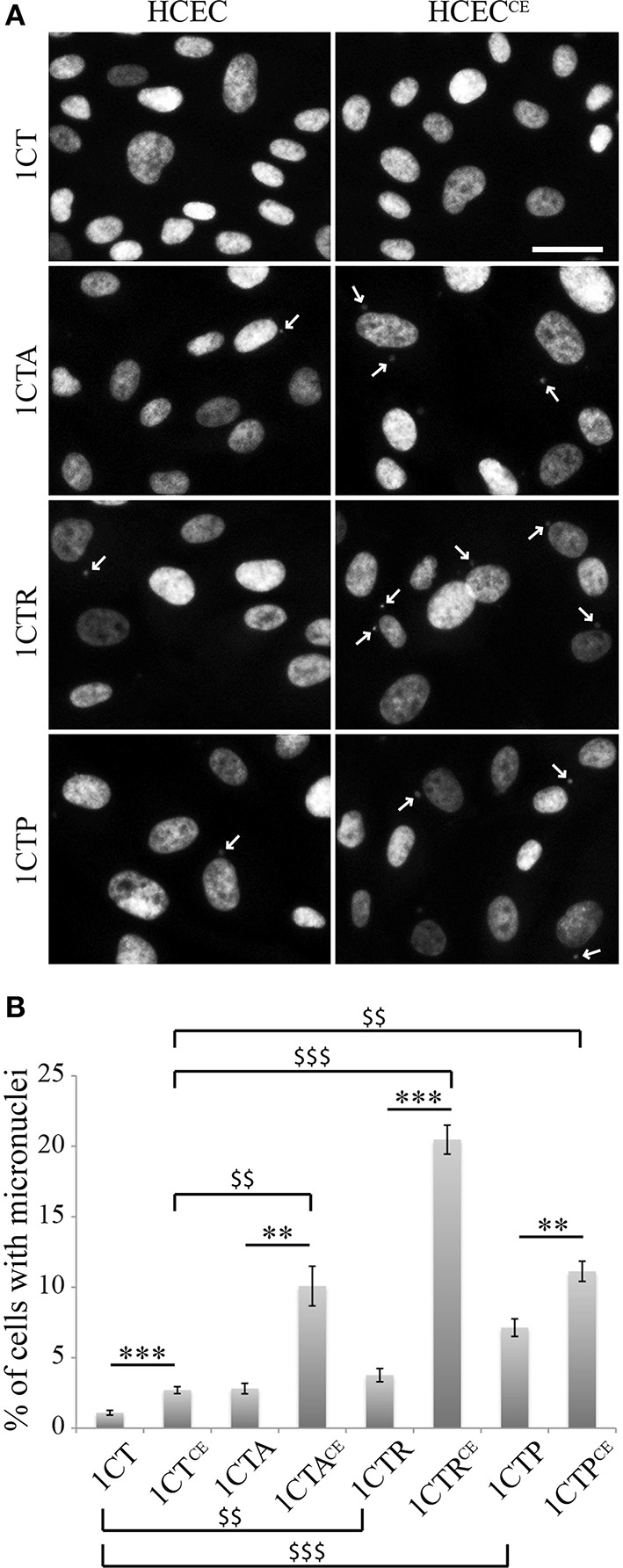
**Micronucleus frequency in HCECs and HCECs^CE^. (A)** Nuclei of HCECs and HCECs^CE^ were stained with DAPI. The frequency of cells with micronuclei (white arrows) was quantified by fluorescence visualization. **(B)** Quantification of HCECs with micronuclei presented in **(A)**. Results represent the mean ± SEM of at least three independent experiments; statistical differences were analyzed by a Student's *t*-test (indicated by asterisks) between the indicated conditions, or by one-way ANOVA (indicated by dollars) for multiple comparisons, followed by Tukey's HSD *post-hoc* test (** or $$ *P* < 0.01; *** or $$$ *P* < 0.001).

Defective DNA repair capacities can generate genetic instabilities, a hallmark of cancer cells. Therefore, the micronucleus frequency was quantified in untreated control cells and cells chronically exposed to EcolCDT (Figure 5). While 1.1% of 1CT cells exhibited micronuclei, CDT chronic exposure induced a 2.5-fold increase of micronucleus-containing cells, representing 2.7% of the entire population in 1CT^CE^ cells. This indicates that the subtoxic dose of EcolCDT used for chronic exposure is yet sufficient to enhance genetic instability. The three 1CT derivative cell lines already present a greater rate of micronucleus-positive cells compared to their parental counterpart. Indeed, the percentage of cells with micronuclei is 2.8% for 1CTA (tendency but not significantly different from 1CT; *P* = 0.0844), 3.75% for 1CTR (*P* < 0.0001), and 7.14% for 1CTP (*P* < 0.001). 1CTA^CE^ cells show a 3.6-fold augmentation of cells with micronuclei compared to 1CTA (*P* < 0.01). Micronucleus induction after chronic exposure to CDT is further enhanced in 1CTR^CE^ that present 5.4 times more cells with micronuclei compared to 1CTR, reaching to approximately 20% of total cells. Finally, albeit 1CTP cells are the most prone to genetic instability, the chronic exposure to CDT only induced a 1.6-fold increase of micronucleus frequency. When analyzing the effect of chronic exposure according to the genotype of the cells, it appears that CDT has a stronger effect in CTA^CE^, CTR^CE^, and CTP^CE^ compared to 1CT^CE^ (*P* < 0.01, < 0.01, and < 0.0001, respectively). In conclusion, the chronic exposure to EcolCDT induces micronucleus formation only modestly in 1CT and 1CTP cells, and this effect is highly potentiated in 1CTA and 1CTR cells.

**Figure 6:**
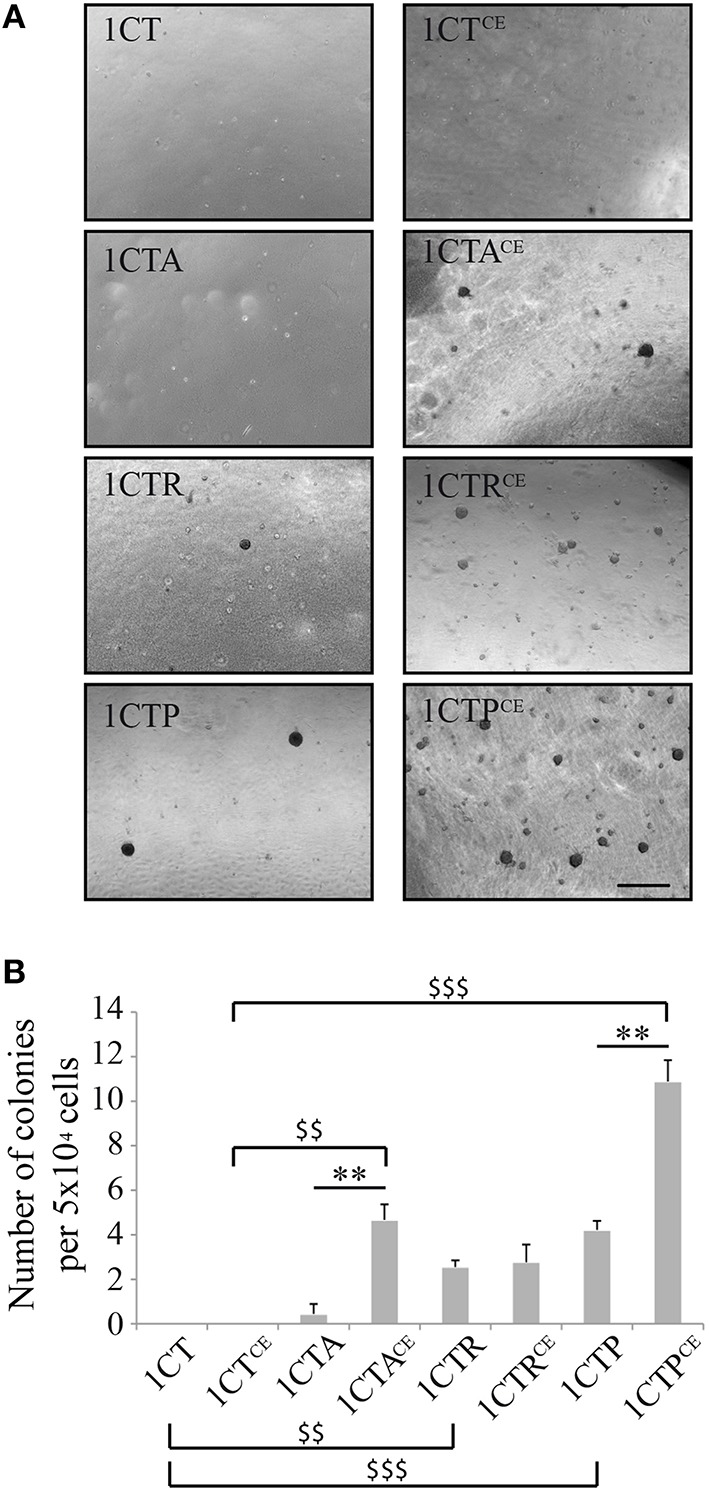
**Chronic exposure to EcolCDT enhances anchorage-independent growth in 1CTA and 1CTP but not in 1CT and 1CTR. (A)** HCECs and HCECs^*CE*^ were cultured in soft-agar in 6-well plates for 3 weeks in triplicates, and colonies larger than 100 μm in size were scored. **(B)** Quantification of the soft-agar assay presented in **(A)**. Results represent the mean ± SEM of three independent experiments; statistical differences were analyzed by a Student's *t*-test (indicated by asterisks) between the indicated conditions, or by one-way ANOVA (indicated by dollars) for multiple comparisons, followed by Tukey's HSD *post-hoc* test (** or $$ *P* < 0.01; $$$ *P* < 0.001).

Normal epithelial cells cannot proliferate without attachment to the extracellular matrix, and must acquire the ability to grow independently of anchorage to become cancer cells. In an attempt to confirm that the genetic instability caused by chronic exposure to EcolCDT constitutes a hallmark of malignant transformation, the soft agar colony assay was conducted in 1CTs vs. 1CTs^CE^ cells (Figure 6). Under our experimental conditions, 1CT cells are not able to form colonies in soft agar even when chronically exposed to CDT. In comparison, 1CTA cells form very few colonies (non-significant compared to 1CT; *P* = 0.7838), and this ability to grow anchorage independently is drastically enhanced after chronic exposure to CDT, to approximately 10-fold the basal 1CTA level. Compared to 1CT, 1CTR, and 1CTP cells exhibit a low but slightly higher number of colonies as previously reported (Eskiocak et al., 2011), with statistical significance (*P* < 0.01 and < 0.0001, respectively, compared to 1CT). However, they present different behavior under chronic exposure condition. Similar to APC deficient cells, 1CTP^CE^ cell ability to grow anchorage independently was statistically increased compared to 1CTP, whereas the number of colonies does not change between 1CTR and 1CTR^CE^. Thus, anchorage independent growth only depends on oncogenic KRAS^V12^ expression in 1CTR^CE^. In 1CTP^CE^, we observed a cumulative effect on colony formation dependent on CDT exposure and p53 downregulation (*P* < 0.001, 1CTP^CE^ vs. 1CT^CE^). According to these results, chronic exposure to EcolCDT cannot by itself promote anchorage-independent growth in normal HCECs, but require particular precancerous-associated genetic alterations in *APC* or *TP53* but not in *KRAS*.

## Discussion

In the last decade, there is mounting evidence directly implicating the human microbiome in carcinogenesis in various organs (Bultman, 2014). The precise role of CDT in bacterial pathogenicity is still not fully understood, but experimental evidences demonstrate in mouse models that production of CDT by *C. jejuni* or *H. hepaticus* is associated with dysplastic changes in their respective niches (Fox et al., 2004; Ge et al., 2007). However, little is known concerning the CDT-mediated cellular defects and the underlying mechanisms that could contribute to malignant transformation. Transgenic rat embryonic fibroblasts and human CRC HCT116 cells acquire hallmarks of cancer after chronic exposure to CDT (Guidi et al., 2013). However, rat embryonic fibroblasts do not represent the primary target of CDT-producing bacteria, and HCT116 are already cancer cells, implying that other cellular models that are more relevant are needed to unravel the mechanism of CDT in the initiation or promotion of malignant transformation. CDT has been shown to be toxic in a broad range of epithelial cells, and many clues indicate that CDT can destabilize the epithelial barrier to promote bacterial colonization (Jinadasa et al., 2011; DiRienzo, 2014). Therefore, we assessed the impact of EcolCDT acute or chronic exposure on normal HCECs obtained from healthy patients (Roig et al., 2010). Moreover, we conducted a comparative study by analyzing isogenic cells, derived from the 1CT lineage and used these as models of three different genetic alterations frequently observed in CRC: loss of APC or p53, and KRAS^V12^ oncogenic activation (Smith et al., 2002). Our results argue for potential a role of EcolCDT in CRC promotion, but not initiation, by perturbing different cellular pathways regarding the precancerous genetic environment.

Our comparative observations of the four HCECs isogenic cell lines reveal differential effects of EcolCDT when critical tumor suppressors or oncogene are deregulated. Basically, EcolCDT displays a dose-dependent cytotoxicity on 1CT cells, associated to DSB induction signaled by γH2AX increase and 53BP1 accumulation at damaged sites, and micronucleus formation. These data are in agreement with the CdtB DNase activity and its associated cellular phenotypes (Bezine et al., 2014). Nevertheless, we do not exclude that the second characterized CdtB PIP3 phosphatase activity (Shenker et al., 2007, 2016) could play a role in the HCEC behavior after intoxication with EcolCDT (see below).

The 1CTA cells are not more sensitive to EcolCDT compared to control cells, and DSB are formed in similar kinetics in both entire cell populations regarding 53BP1 immunofluorescence (Figure 2). However, the CDT-induced γH2AX increase observed by In-Cell Western is greater in 1CTA than in 1CT (Figure 3C). We previously reported that CDT-mediated DSB formation increases over time in a cell cycle-dependent manner (Fedor et al., 2013). As 1CT and 1CTA exhibit the same proportion of damaged cells after CDT treatments, this suggests that their proliferation status is similar. Then, the more pronounced γH2AX increase in 1CTA might reflect a defect in DNA repair with loss of APC function. Indeed, the repair of etoposide-induced DNA damage was less efficient in APC-defective cells. APC has been implicated in the regulation of Base Excision Repair, and thus of single strand breaks repair (Jaiswal et al., 2006). Our previous work strongly suggests that CDT generates both DSBs and single strand breaks (Fedor et al., 2013), establishing a possible link between CDT genotoxicity and APC function. Moreover, APC ensures high-fidelity chromosome segregation, especially in combination with aneugenic stresses, and chromosome mis-segregation in APC deficient cells is most probably associated with DNA damage (Poulton et al., 2013). CDT induces micronuclei even in the presence of APC, suggesting that the enhanced chromosome segregation defects in CDT-exposed 1CTA cells is at least in part responsible for γH2AX accumulation. Implication of p53 in the response to EcolCDT-induced DNA damage was not surprising since it has a well-defined role in DNA damage responses (Toledo and Wahl, 2006). Our data support that both 53BP1 accumulation and γH2AX increase after CDT or etoposide are altered in 1CTP cells, demonstrating that activation of the DNA damage response by CDT is impaired through p53 loss in HCEC. In other words, our results support that the loss of the tumor suppressors APC or p53 sensitize HCECs to CDT-induced genotoxicity.

In contrast, 1CTR cells do not show any defect in 53BP1 foci formation or in γH2AX induction in response to CDT, suggesting that DSB signaling and repair is not affected by KRAS oncogenic activation. Moreover, 1CTR are more resistant to CDT than the three other HCECs. If one assumes that DNA damage is not responsible for the different CDT-mediated cellular outcomes between 1CTR and 1CT, then it should rely on CdtB phosphatase activity. CdtB hydrolyses PIP3 to phosphatidylinositol-3,4-biphosphate and thus alters the PI-3K/PIP3/Akt/ pGSK3β signaling pathway (Shenker et al., 2007, 2016). Intracellular PIP3 is primarily synthetized by PI-3Ks and regulates a variety of physiological processes including cell proliferation, cell survival, and intracellular vesicle trafficking (Manna and Jain, 2015). Interestingly, PI-3K is one of the main effector of KRAS, suggesting that the oncogenic activation of KRAS in 1CTR cells enhance the pool of intracellular PIP3, the substrate of the CdtB phosphatase activity. According to the vast spectrum of processes regulated by phosphoinositides (Sasaki et al., 2007), it is therefore tempting to speculate that the concomitant PI-3K overactivation and CdtB phosphatase activity in 1CTR exposed to CDT will affect numerous downstream physiological functions.

Thus, chronic exposure to EcolCDT should induce distinct cellular defects in the four HCECs cell lines, especially concerning malignant transformation. 1CT cells, considered as normal HCEC, only display modest changes after being chronically exposed to EcolCDT. Indeed, albeit micronucleus frequency slightly increased in 1CT^CE^ compared to 1CT, they show identical DNA damage responses and are not able to grow anchorage independently. This suggests that chronic exposure of normal HCEC to EcolCDT is not a potent initiator of CRC. In contrast, our observations indicate that 1CTA^CE^ acquire different hallmarks of cancer, as they display an altered DNA damage response and a greater enhancement of chromosomal instability and anchorage-independent growth. These cellular phenotypes may be a consequence of APC loss-related sensitization to the DNA damage induced by CDT. Alternatively, it may be due to the cumulative effect of APC deficiency and CDT-induced DSBs on cytoskeleton, both of which destabilizing the actin network (Fearnhead et al., 2001; Frisan et al., 2003; Supplementary Figure 1C). 1CTR and 1CTP cells already exhibit signs of malignant transformation, with spontaneous micronuclei formation and the ability to grow anchorage independently. Furthermore, p53 deficiency intrinsically perturbs the DNA damage response (Toledo and Wahl, 2006). CDT chronic exposure augments anchorage-independent growth of 1CTP but has limited effects on chromosomal instability. Importantly the micronucleus basal level is relatively high in 1CTP, probably due to the genotoxic stresses inherent to the cell culture conditions. The CDT-mediated effects on chromosome mis-segregation might thus be partially masked. In contrast, 1CTR ability to grow anchorage independently is not impacted by chronic exposure to CDT, whereas micronucleus frequency dramatically increased compared to all other cell lines. These data suggest that the cumulative effects of PI-3K overactivation induced by KRAS^V12^ expression and of CdtB phosphatase activity modify the phosphoinositide pool content in a way that will ultimately affect chromosome segregation. Deciphering the molecular mechanisms that drive these genetic alterations will be of great interest to gain a better understanding of the cellular impact of CdtB phosphatase activity on the tumor microenvironment involving PI-3K pathway alterations.

The Fearon and Vogelstein genetic model of CRC carcinogenesis predicts the sequential mutation of specific genes initiated by the mutations of the *APC* gene followed by mutations on *KRAS* and on *TP53* (Fearon and Vogelstein, 1990). However, a genetic study of a CRC patient cohort revealed that these mutations could occur independently in tumors, especially the association of mutations in *TP53* and *KRAS* (Smith et al., 2002). More generally, the combinations of KRAS mutation with APC and/or TP53 are represented in only 20% of the CRC tumors. In contrast, the most common combination of mutations affected *APC* and *TP53*.

Interestingly, these observations correlate with our findings that EcolCDT may affect 1CTR through a mechanism different from 1CTA and 1CTP.

Overall, these results strongly suggest that EcolCDT cannot efficiently initiate CRC carcinogenesis by itself. To support this statement, CDT production by *C. jejuni* or *H. hepaticus* has been associated to dysplasia only after infection of NF-κB-deficient mice or liver disease-susceptible mice, respectively (Fox et al., 2004; Ge et al., 2007). The present study favors a role of EcolCDT in CRC promotion in genetically altered precancerous HCECs. *APC* gene mutations are found in 50–80% of sporadic CRC and are considered as early initiating events (Fearnhead et al., 2001). Intriguingly, our observations indicate that the more pronounced malignant transformation after chronic exposure to EcolCDT concerned the APC-deficient HCEC. Therefore, CDT production by pathogenic bacteria should be an aggravating factor in a majority of CRC susceptible genetic backgrounds. Besides, the alternative genetic pathways may also be affected, at least those implying *TP53* or *KRAS* mutations. Finally, CDT does not represent the unique virulent factor produced by pathogenic *E. coli*. CDT association to CRC has been observed from *E. coli* strains harboring other cell cycle destabilizing toxins (Buc et al., 2013; Bonnet et al., 2014), strengthening the postulate that CDT incidence in CRC is a part of a multifactorial process.

## Author contributions

LH, GM, and JV designed the study. VG, LH, GM, and JV wrote the final manuscript. VG, ID, and JD performed the experiments. All authors read the manuscript and discussed the results.

## Funding

This work was supported by an ANR program to GM (Grant number ANR-10-CESA-011) and by the INRA support to LH and JV (Internal program of the TOXALIM Institute termed collaborative project). NASA grants NNX15A121G and NNX16AE08G (JWS).

### Conflict of interest statement

The authors declare that the research was conducted in the absence of any commercial or financial relationships that could be construed as a potential conflict of interest.
