# Supplementary material for: Genotoxicity of Cytolethal Distending Toxin (CDT) on Isogenic Human Colorectal Cell Lines: Potential Promoting Effects for Colorectal Carcinogenesis
Source: Front Cell Infect Microbiol. 2016 Mar 23;6:34. doi: 10.3389/fcimb.2016.00034 (PMC4803749; doi:10.3389/fcimb.2016.00034)
Supplement: Supplementary file 1 [file DataSheet1.docx]

Supplementary Material

**Genotoxicity of Cytolethal Distending Toxin (CDT) on isogenic human colorectal cell lines: potential promoting effects for colorectal carcinogenesis**

Vanessa Graillot^1$^, Inge Dormoy^1$^, Jacques Dupuy^1^, Jerry W. Shay^2^, Laurence Huc^1^*, Gladys Mirey^1^, Julien Vignard^1^*

^$^: co- first authors

*Correspondence: Julien Vignard: [julien.vignard@toulouse.inra.fr](mailto:julien.vignard@toulouse.inra.fr)

Laurence Huc: laurence.huc@toulouse.inra.fr

# Supplementary Figures and Tables

## Supplementary Figures

**Supplementary Figure 1.** Characterization of the 1CTA cell line. **(A)** Whole total cellular lysates were loaded in SDS-PAGE. Western blot were performed using anti-APC and anti-Lamin A/C as loading control. **(B)** Beta-catenin protein was localized in 1CT and 1CTA cells under basal conditions. DAPI was used to stain nuclei. Images were acquired by confocal imaging according to a stacking mode. The pictures presented beta-catenin (green) in the same confocal plan than nuclei (blue). **(C)** Actin was localized in 1CT and 1CTA cells under basal conditions. DAPI was used to stain nuclei. Images were acquired by confocal imaging according to a stacking mode. The pictures presented actin (red) in the same confocal plan than nuclei (blue). Scale bars = 10µm.

## Supplementary Tables

|  | ***STR locus*** | | | | | | | | | |
| --- | --- | --- | --- | --- | --- | --- | --- | --- | --- | --- |
| ***cell lines*** | ***Amel*** | ***CSF1PO*** | ***D13S317*** | ***D16S539*** | ***D21S11*** | ***D5S818*** | ***D7S820*** | ***TH01*** | ***TPOX*** | ***vWA*** |
| ***CT*** | X,Y | 10, 12 | 12, 13 | 10, 11 | 28 | 11, 14 | 9, 11 | 9.3 | 11 | 16, 18 |
| ***CTA*** | X,Y | 10, 12 | 12, 13 | 10, 11 | 28 | 11, 14 | 9, 11 | 9.3 | 11 | 16, 18 |
| ***CTR*** | X,Y | 10, 12 | 12, 13 | 10, 11 | 28 | 11, 14 | 9, 11 | 9.3 | 11 | 16, 18 |
| ***CTP*** | X,Y | 10, 12 | 12, 13 | 10, 11 | 28 | 11, 14 | 9, 11 | 9.3 | 11 | 16, 18 |

**Supplementary Table 1.** STR Profiles of HCECs. All the cell lines were submitted to a STR profiling on 9 loci: THO1, TPOX, vWA, CSF1PO, D16S539, D7S820, D13S317, *D21S11*, D5S818 and the sex chromosome marker Amelogenin (Amel). The data were analyzed by using Gene Mapper ID-X software to categorize peaks according to their size in relation to an internal standard run.
